# Transcriptome profiling of mice testes following low dose irradiation

**DOI:** 10.1186/1477-7827-11-50

**Published:** 2013-05-28

**Authors:** Kirstine C Belling, Masami Tanaka, Marlene Danner Dalgaard, John Erik Nielsen, Henrik Bjørn Nielsen, Søren Brunak, Kristian Almstrup, Henrik Leffers

**Affiliations:** 1 Center for Biological Sequence Analysis, Department of Systems Biology, Technical University of Denmark, 2800 Lyngby, Denmark; 2 Department of Nutrition, Junior College Division, The University of Aizu, Aizu-Wakamatsu 965-8570 Japan; 3 Department of Pharmacology, St. Marianna University School of Medicine, Kawasaki 216-8511 Japan; 4 Department of Growth and Reproduction, Rigshospitalet, 2100 Copenhagen, Denmark; 5 Department of Disease Systems Biology, Faculty of Health Sciences, Novo Nordisk Foundation Center for Protein Research, University of Copenhagen, Blegdamsvej 3A, 2200 Copenhagen, Denmark

**Keywords:** Mice, Testis, Irradiation, Gene expression, Transcriptomics, Microarray, Clustering, Leydig cell, Hyperplasia

## Abstract

**Background:**

Radiotherapy is used routinely to treat testicular cancer. Testicular cells vary in radio-sensitivity and the aim of this study was to investigate cellular and molecular changes caused by low dose irradiation of mice testis and to identify transcripts from different cell types in the adult testis.

**Methods:**

Transcriptome profiling was performed on total RNA from testes sampled at various time points (n = 17) after 1 Gy of irradiation. Transcripts displaying large overall expression changes during the time series, but small expression changes between neighbouring time points were selected for further analysis. These transcripts were separated into clusters and their cellular origin was determined. Immunohistochemistry and *in silico* quantification was further used to study cellular changes post-irradiation (pi).

**Results:**

We identified a subset of transcripts (n = 988) where changes in expression pi can be explained by changes in cellularity. We separated the transcripts into five unique clusters that we associated with spermatogonia, spermatocytes, early spermatids, late spermatids and somatic cells, respectively. Transcripts in the somatic cell cluster showed large changes in expression pi, mainly caused by changes in cellularity. Further investigations revealed that the low dose irradiation seemed to cause Leydig cell hyperplasia, which contributed to the detected expression changes in the somatic cell cluster.

**Conclusions:**

The five clusters represent gene expression in distinct cell types of the adult testis. We observed large expression changes in the somatic cell profile, which mainly could be attributed to changes in cellularity, but hyperplasia of Leydig cells may also play a role. We speculate that the possible hyperplasia may be caused by lower testosterone production and inadequate inhibin signalling due to missing germ cells.

## Background

Radiotherapy is used routinely to treat testicular cancer. Cells in the testis display different radio-sensitivity and the degree of radiation-induced damage depends on dose and fractionation of the radiation [1]. Some cells are very sensitive and undergo apoptosis after low dose irradiation, others are only partially affected and presumably recover after a period of time, while yet others seem completely unaffected. Spermatogonial stem cells (SSCs) are the most radio-resistant germ cells, probably due to their slow turnover. Contrarily, A spermatogonia have the highest turnover and are the germ cells most susceptible to irradiation-induced damage [2,3] followed by Intermediate and B spermatogonia [4,5]. The more differentiated germ cells such as spermatocytes and spermatids seem unaffected by low dose irradiation and continue their maturation toward spermatozoa [1].

The somatic cells in the testis are also to some extent affected by irradiation. Human Leydig cells are damaged by the irradiation doses used to treat testicular cancer patients (16–20 Gy) [1,6,7], although in many cases the testosterone production is maintained. Rodent Leydig cells have shown dysfunction after high dose irradiation (5 Gy) leading to low testosterone and increased serum luteinizing hormone (LH) levels [8]. Similarly, it has also been reported that rodent Sertoli cells are affected by high dose irradiation (5 Gy) [9].

Low dose irradiation of adult rodent testes kills A through B spermatogonia, which creates a “gap” in the germinal epithelium [10]. The gap moves through the differentiation stages of the germ cells as spermatogenesis continues and SSCs re-populate the testis. The cellular and molecular changes caused by the movement of the gap can be used to study gene expression in adult spermatogenesis. However, by studying the whole testis we investigate changes in the relative gene expression, i.e. the contribution from each cell type to the total RNA pool, which is determined by the concentration of the RNA in the specific cell type and the percentage of the total volume that the cell type occupies - the cellularity [11].

In this study, we identified five clusters of cell type-specific transcripts by transcriptome profiling. The transcripts in these clusters all had gene expression profiles that could be explained by changes in the cellularity during recovery from irradiation. We observed large expression changes in the somatic cell cluster and further investigations by immunohistochemistry (IHC) revealed that low dose irradiation seemed to cause hyperplasia of the Leydig cells, whereas peritubular myoid (PTM) and Sertoli cells seemed unaffected.

## Methods

### Mice testis preparation

Treatment of mice and preparation of testicular RNA and sections were performed as in Shah et al., [10]. In brief, male C3H/He mice from Japan SLC (Shizuoka, Japan) were maintained under controlled conditions (22 ± 2°C, 55 ± 5% humidity, 12 h light/dark cycle, lights on 0600 h) with laboratory chow (CE-2, Japan Crea, Tokyo, Japan) and water *ad libitum*. Eleven-week old mice were anesthetised with pentobarbital and covered with lead sheeting except for the scrotum. The testes were locally exposed to X-ray irradiation with 1 Gy. Testes from one to four mice were sampled and weighted regularly during recovery on days 0, 3, 7, 10, 14, 17, 21, 24, 28, 31, 35, 38, 42, 45, 48, 52, 56, and 59. One testis was fixed in 4% paraformaldehyde in 0.1 M phosphate buffer, pH 7.4, overnight at 4°C and subsequently dehydrated in graded series of ethanol and embedded in paraffin for IHC. The contralateral testis was snap-frozen in liquid nitrogen for later preparation of total RNA.

The Japanese Pharmacological Society approved the animal study. The animals were treated according to generally accepted guidelines for animal experimentation at St. Marianna University Graduate School of Medicine and guiding principles for the care and use of laboratory animals.

### Gene expression microarrays

Total RNA was purified from whole testis samples collected pi day 3 to day 59 (n = 17) with NucleoSpin RNA II (Macherey-Nagel, Dueren, Germany) according to the manufacturer’s protocol. RNA quality was determined using Bioanalyzer nano kit (Agilent Technologies, Santa Clara, California, US). The samples were amplified (one round) using the MessageAmp II aRNA Amplification Kit (Applied Biosystems, Carlsbad, California, US) and the aRNA was applied to Agilent whole mouse genome 4 × 44K oligo microarrays. Hybridisation and scanning of the one-colour arrays were done as described by the manufacturer (Agilent Technologies, Santa Clara, California, US). The microarray data analysis was performed in the R software [12] where the gProcessedSignals were loaded into the limma R/Bioconductor package [13,14] and normalised between arrays using the quantile normalisation method [15]. The probes were collapsed for each systematic transcript ID by the median expression value.

### Enrichment score

Transcripts with expression profiles potentially explained by changes in cellularity due to the recovery from irradiation were identified and selected for further analysis based on the enrichment score (ES) calculated as follows:

$$\text{Enrichment}\;\text{score}\;\left(\mathrm{ES}\right)=\frac{\sum_{i=1}^{\left(n-1\right)}\left|GE_{i}-GE_{\left(i+1\right)}\right|}{\sum_{i=1}^{n}{\left(GE_{i}-\overset{―}{\mathrm{GE}}\right)}^{2}}$$

where *GE*_*i *_and *GE*_*(i + 1) *_is the expression value for a transcript at time point *i* and the neighbouring time point *i* + 1, respectively, and $\overset{―}{\mathrm{GE}}$ is the mean expression value for a transcript through the time series.

The false discovery rate (FDR) of the ES was calculated for each transcript based on ten calculations on shuffled time points. A subset of transcripts was chosen for further analysis all with a cumulative minimum of the FDR ≤ 30% and a standard deviation (SD) of the most extreme expression value in the time series ≤ 15%.

### Cluster analysis

Cluster analyses were performed on the subset of transcripts selected as described above. A distance matrix was generated with the pair-wise correlation variances of the gene expression values during the time series centred around 1. The transcripts were clustered according to the distance matrix using partitioning around medoids (PAM) clustering, which was performed multiple times with different number of clusters. The number of clusters that separated most unique transcript clusters was chosen as the best separation.

### Association of transcript clusters to specific testis cells

To determine the cellular origin of the transcript clusters, we compared their gene expression patterns to the expression profiles of cell markers determined in our previous study of the same testis samples where we used differential display and *in situ* hybridisation [10]. We also mapped testicular cell-specific markers to the clusters to confirm their cellular origin [16-18].

### Gene set enrichment analysis

Gene set enrichment analysis (GSEA) was performed on the transcripts in each cluster separately using DAVID [19,20]. All genes represented on the array were used as background. The cut off for statistical significance was set to the Bonferroni corrected p-value ≤ 0.01.

### Immunohistochemistry (IHC)

The following primary antibodies were used: Vimentin/HRP (Dako, Glostrup, Denmark; U7034), Transforming growth factor β receptor III (Tgfbr3) 1:75 (Santa Cruz Biotechnology, Santa Cruz, CA, USA; sc-6199), 3β-hydroxysteroid-dehydrogenase (Hsd3b) 1:6000 (R1484 a gift from Prof. J. Ian Mason, Edinburgh), and Smooth muscle actin (SMA) 1:600 Abcam ab5694 (330 Cambridge Science Park, Cambridge CB4 OFL, UK).

Vimentin was used according to the manufacturer’s protocol. In short, the sections were deparaffinised, rehydrated and blocked for endogen peroxidase with H_2_O_2_, washed in tap water, placed 5 min in TBS (0.5 M Tris/HCl, 0.15 M NaCl, pH 7.6) at 37°C, incubated with 1:10 Trypsin (Gibson 15400) in TBS 15 min at 37°C, and exposed to the antibody for 1 h at room temperature. Development was performed with 3-amino-9-ethylcarbazole (AEC). Thorough washing with TBS was performed after each individual step and finally the sections were washed in water before a short staining with Meyers haematoxylin.

The three remaining antibodies, against Tgfbr3, Hsd3b and SMA, were used as in a protocol based on a Zymed histostain kit (Invitrogen, Carlsbad, CA, USA). In short, the sections were deparaffinised, rehydrated and blocked for endogen peroxidase as described above, followed by microwave treatment for 15 min in TEG buffer (10 mM Tris, 0.5 mM EGTA, pH 9.0) for Tgfbr3 and Hsd3b, whereas Citrate buffer (10 mM, pH 6) was used for SMA. Cross reactivity of the antibodies was minimized by treatment with 0.5% milk powder diluted in TBS. Sections were exposed to the primary antibodies over night at 5°C and 1 h at room temperature, then incubated with biotinylated goat anti-rabbit IgG or with biotinylated donkey anti-goat IgG 1:400 in TBS (The binding site Ltd., Birmingham, UK; AB360) for Tgfbr3 before exposure to a peroxidase-conjugated streptavidin complex. Finally, the sections were developed with AEC and counter stained with Meyers haematoxylin. Thorough washing with TBS was performed after each individual step. Slides without addition of primary antibodies were used as controls.

### *In silico* quantification of Leydig cells

In order to obtain a measure of the amount of Leydig cells relative to other cells in the testis, we quantified the area covered by Leydig cells relative to the area covered by other testicular cells. Three different areas, each representing more than ten seminiferous tubules, from sections of the testis of representative days pi were stained with the Leydig cell marker Hsd3b, scanned and evaluated *in silico* with the VisioMorph add-in in the VIS program (Visiopharm A/S, Hørsholm, Denmark). A red colour was assigned to the red Hsd3b staining, blue to other cells (Meyers haematoxylin) and white to the background (see Additional file 1: Figure S1). A Baysian classification of the colour space from the stained sections was applied and subsequently red areas smaller than 20 μm^2^ assigned as blue. The areas of red, blue and white were measured and the ratio of red to blue used as a measure of the amount of Leydig cells relative to the amount of other testis cells.

## Results

### Clusters of transcripts affected by low dose irradiation

Gene expression profiling was performed on total RNA from mice testis (n = 17) from a time series following low dose irradiation (1 Gy). In total, 988 transcripts were selected for further analysis. These transcripts all had expression profiles during the time series that potentially were caused by the recovery from irradiation, i.e. transcripts with large overall expression changes during the time series, but small expression changes between neighbouring time points. We performed PAM clustering of the distance matrix of the pair-wise correlation variances and identified five clusters with unique expression patterns. This was the maximum number of clusters separable in this data set. The names of the transcripts in each cluster are available in additional material (see Additional file 2: Table S1, Additional file 3: Table S2, Additional file 4: Table S3, Additional file 5: Table S4, Additional file 6: Table S5).

### Assignment of transcript clusters to specific testicular cells

To identify the cellular origin of the clusters, we compared the expression patterns of the five clusters with the expression patterns of cell markers investigated in our previous study of the same testicular material [10]. We found that four of the clusters identified in this study independently had similar expression profiles as four cell-specific markers identified in our previous study. The four markers were in our previous study associated with spermatogonia (cluster 1 in the current study; n = 109), spermatocytes (cluster 2; n = 164), spermatids (cluster 3; n = 246) and Sertoli cells (cluster 5; n = 196). Since transcripts in the early spermatid cluster showed a decrease in expression with a trough already at pi day 24, we assigned the fifth novel cluster, with transcripts that showed a decrease with a trough in expression at pi day 27, to late spermatids (cluster 4; n = 273). The expression level of most transcripts returned to pre-irradiation levels around pi day 40 where a complete round of spermatogenesis had taken place [21].

**Figure 1:**
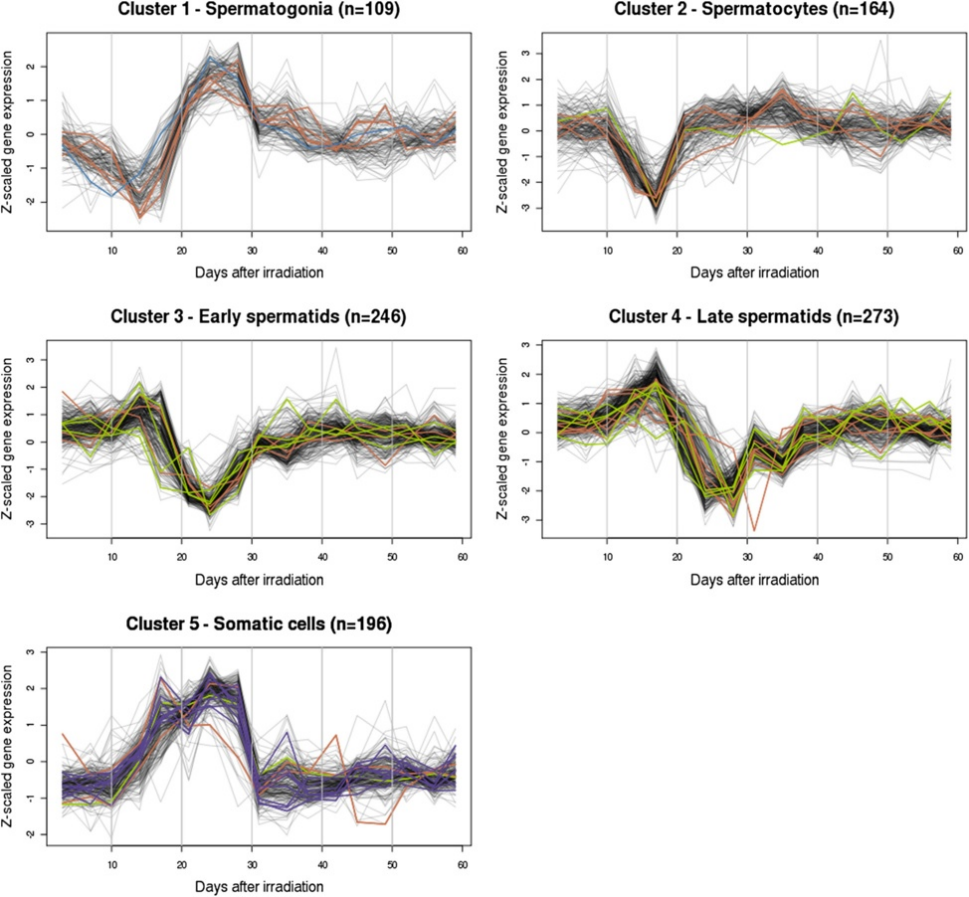
**Gene expression profiles of the five transcript clusters during recovery from irradiation.** The clusters contain the subset of 988 transcripts changing in expression during recovery from irradiation, which was separated into clusters by PAM. The number of transcripts in each cluster is stated in the title of each plot. The cell specific markers are coloured as following: Blue: Spermatogonia; Red: Spermatocytes; Green: Spermatids; Purple: Somatic cells.

**Figure 2:**
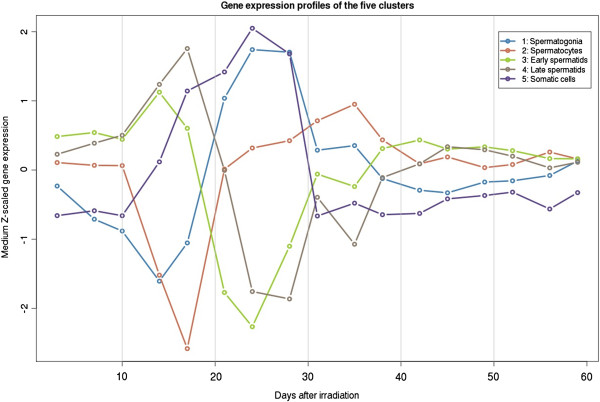
**The median z-scaled gene expression of the five transcript clusters during recovery from irradiation.** The clusters are coloured as following: Blue: Spermatogonia (cluster 1); Red: Spermatocytes (cluster 2); Green: Early spermatids (cluster 3); Brown: Late spermatids (cluster 4); Purple: Somatic cells (cluster 5).

To add further evidence of the association of cell types to the clusters, we identified previously published cell-specific markers in the five clusters [16-18]. In total, 39 markers were identified in the five clusters representing spermatogonia (n = 1), spermatocytes (n = 19), spermatids (n = 11), Leydig cells (n = 3), PTM cells (n = 1), and Sertoli cells (n = 4) (Table 1, Figure 1 and Figure 2). The single marker associated with spermatogonia was found in the corresponding cluster (cluster 1). The spermatocyte markers were evenly distributed in all five clusters, but comparing the expression patterns to the profiles identified earlier [10,16] strongly indicated that this cluster (cluster 2) with a reduced expression at pi day 17, mainly contained transcripts from spermatocytes. Most spermatid markers were present in the two clusters that we associated with early and late spermatids (cluster 3 and 4). The transcripts in the somatic cell cluster contained all somatic cell markers (cluster 5), which confirmed that the cluster represented all somatic cells and not only transcripts from Sertoli cells as suggested in our earlier study [10].

**Table 1:** Testis cell-specific markers mapped to the transcript clusters

**Gene symbol**	**Gene name**	**Associated cell**	**Identified cluster #**
Dazl	Deleted in azoospermia-like	Spermatogonia	1
1500011H22Rik	RIKEN cDNA 1500011H22 gene	Spermatocytes	2
1700029G01Rik	RIKEN cDNA 1700029G01 gene	Spermatocytes	3
2410022L05Rik	RIKEN cDNA 2410022 L05 gene	Spermatocytes	2
Akap12	A kinase (PRKA) anchor protein (gravin) 12	Spermatocytes	5
Aldoa-ps1	Aldolase 1, A isoform, retrogene 2	Spermatocytes	4
Atl3	Atlastin GTPase 3	Spermatocytes	4
Cypt3	Cysteine-rich perinuclear theca 3	Spermatocytes	3
D030056L22Rik	RIKEN cDNA D030056L22 gene	Spermatocytes	1
Gsg2	Germ cell-specific gene 2	Spermatocytes	3
H3f3b	H3 histone, family 3B	Spermatocytes	1
Hnrpa2b1	Heterogeneous nuclear ribonucleoprotein A2/B1	Spermatocytes	1
Lyar	Ly1 antibody reactive clone	Spermatocytes	2
Nt5c1b	5′-nucleotidase, cytosolic IB	Spermatocytes	4
Pgk2	Phosphoglycerate kinase 2	Spermatocytes	4
Slc2a3	Solute carrier family 2 (facilitated glucose transporter), member 3	Spermatocytes	2
Spert	Spermatid associated	Spermatocytes	4
Stard10	START domain containing 10	Spermatocytes	4
Stmn1	Stathmin 1	Spermatocytes	1
Vkorc1	Vitamin K epoxide reductase complex, subunit 1	Spermatocytes	5
Acrv1	Acrosomal vesicle protein 1	Spermatids	3
Actl7a	Actin-like 7a	Spermatids	4
Akap4	A kinase (PRKA) anchor protein 4	Spermatids	4
Marcksl1	MARCKS-like 1	Spermatids	5
Pdpk1	3-phosphoinositide dependent protein kinase-1	Spermatids	4
Pdzk1	PDZ domain containing 1	Spermatids	4
Prm1	Protamine 1	Spermatids	2
Spag4l	Sperm associated antigen 4-like	Spermatids	3
Tctex1d1	Tctex1 domain containing 1	Spermatids	4
Tnp2	Transition protein 2	Spermatids	3
Zpbp	Zona pellucida binding protein	Spermatids	3
Hsd17b3	Hydroxysteroid (17-beta) dehydrogenase 3	Leydig cells	5
Hsd3b1	Hydroxy-delta-5-steroid dehydrogenase, 3 beta- and steroid delta-isomerase 1	Leydig cells	5
Cyp17a1	Cytochrome P450, family 17, subfamily a, polypeptide 1	Leydig cells	5
Acta2	Actin, alpha 2, smooth muscle, aorta	PTM cells	5
Cldn11	Claudin 11	Sertoli cells	5
Clu	Clusterin	Sertoli cells	5
Ctsl	Cathepsin L	Sertoli cells	5
Vim	Vimentin	Sertoli cells	5

The table lists the 39 previously published cell-specific markers used to identify the cellular origin of the transcripts clusters [16-18].

### Gene set enrichment analysis

GSEA was performed on the genes from each cluster separately to gain knowledge of overrepresented functional categories in each cluster. Statistical significant categories are listed in Table 2. Of particular interest, this analysis revealed overrepresentation of genes involved in mRNA processing, spliceosome and nucleus for the transcripts in the spermatogonia cluster. The transcripts in the spermatocyte cluster were enriched in male gamete generation, spermatogenesis and cell cycle process. The early spermatid cluster differed from the late spermatid cluster in enrichment for acrosomal vesicle. The somatic cell cluster was enriched in processes such as lipid metabolic process, response to oxidative stress and lipid biosynthetic process. All enriched Gene Ontology categories are available in the additional material (see Additional file 7: Table S6, Additional file 8: Table S7, Additional file 9: Table S8, Additional file 10: Table S9 and Additional file 11: Table S10).

**Table 2:** **Significant categories from the GSEA for each transcript clusters performed using DAVID**[19,20]

**Cluster**	**Category**	**Term**	**Count**	**%**	**p-value**	**Fold enrichment**	**Bonferroni p-value**
1: Spermatogonia	BP	mRNA processing	11	11.83	6.12E-07	8.22	3.11E-04
		mRNA metabolic process	11	11.83	2.46E-06	7.05	0.001
		RNA splicing	9	9.68	6.05E-06	8,87	0.003
	CC	Ribonucleoprotein complex	19	20.43	4.15E-12	8.28	6.56E-10
		Macromolecular complex	34	36.56	5.83E-09	2.75	9.21E-07
		Intracellular organelle	60	64.52	2.24E-08	1.54	3.54E-06
		Organelle	60	64.52	2.32E-08	1.54	3.67E-06
		Intracellular part	64	68.82	4.10E-08	1.41	6.48E-06
		Intracellular	64	68.82	2.70E-07	1.36	4.26E-05
		Intracellular organelle part	35	37.63	5.02E-07	2.25	7.93E-05
		Organelle part	35	37.63	5.95E-07	2.24	9.40E-05
		Spliceosome	8	8.60	2.23E-06	12.97	3.53E-04
		Nucleus	38	40.86	1.60E-05	1.85	0.003
		Intracellular membrane-bounded organelle	51	54.84	4.71E-05	1.47	0.007
		Membrane-bounded organelle	51	54.84	4.90E-05	1.47	0.008
	MF	RNA binding	21	22.58	2.81E-12	7.11	4.35E-10
		Nucleic acid binding	31	33.33	1.36E-06	2.36	2.11E-04
2: Spermatocytes	BP	Sexual reproduction	18	12.41	1.02E-09	6.70	7.80E-07
		Gamete generation	16	11.03	7.42E-09	6.94	5.67E-06
		Male gamete generation	14	9.66	2.03E-08	7.89	1.55E-05
		Spermatogenesis	14	9.66	2.03E-08	7.89	1.55E-05
		Multicellular organism reproduction	16	11.03	1.22E-07	5.62	9.34E-05
		Reproductive process in a multicellular organism	16	11.03	1.22E-07	5.62	9.34E-05
		Reproductive process	18	12.41	1.01E-06	4.17	7.72E-04
		Reproduction	18	12.41	1.11E-06	4.14	8.44E-04
		Cell cycle process	14	9.66	2.88E-06	5.12	0.002
	CC	Intracellular organelle part	45	31.03	1.01E-06	2.03	2.04E-04
		Macromolecular complex	39	26.90	1.16E-06	2.21	2.36E-04
		Organelle part	45	31.03	1.22E-06	2.02	2.48E-04
		Intracellular part	90	62.09	1.67E-06	1.35	3.39E-04
		Intracellular membrane-bounded organelle	74	51.03	6.04E-06	1.47	0.001
		Membrane-bounded organelle	74	51.03	6.04E-06	1.47	0.001
		Intracellular organelle	80	55.17	6.71E-06	1.41	0.001
		Organelle	80	55.17	6.87E-06	1.41	0.001
3: Early spermatids	BP	Sexual reproduction	19	9.60	2.17E-10	6.69	1.19E-07
		Male gamete generation	13	6.57	2.33E-07	7.03	1.28E-04
		Spermatogenesis	13	6.57	2.33E-07	7.03	1.28E-04
		Reproductive process	19	9.60	3.10E-07	4.21	1.70E-04
		Reproduction	19	9.60	3.51E-07	4.17	1.93E-04
		Gamete generation	14	7.07	5.99E-07	5.81	3.29E-04
		Multicellular organism reproduction	14	7.07	6.43E-06	4.69	0.004
		Reproductive process in a multicellular organism	14	7.07	6.43E-06	4.69	0.004
	CC	Acrosomal vesicle	10	5.05	1.06E-10	25.52	1.76E-08
		Secretory granule	10	5.05	1.11E-06	9.26	1.84E-04
4: Late spermatids	BP	Sexual reproduction	16	7.24	4.98E-08	6.01	2.95E-05
		Spermatogenesis	12	5.43	1.27E-06	6.83	7.51E-04
		Male gamete generation	12	5.43	1.27E-06	6.83	7.51E-04
		Gamete generation	13	5.88	2.51E-06	5.70	0.001
		Reproductive process in a multicellular organism	14	6.33	3.97E-06	4.97	0.002
		Multicellular organism reproduction	14	6.33	3.97E-06	4.97	0.002
	CC	Cytoplasm	73	33.03	1.65E-05	1.49	0.002
5: Somatic cells	BP	Oxidation reduction	28	15.56	2.32E-09	3.87	3.34E-06
		Lipid metabolic process	28	15.56	4.93E-09	3.74	7.10E-06
		Response to oxidative stress	10	5.56	3.62E-07	10.68	5.22E-04
		Lipid biosynthetic process	16	8.89	4.00E-07	5.21	5.76E-04
		Cellular ketone metabolic process	20	11.11	1.23E-06	3.79	0.002
		Oxoacid metabolic process	19	10.56	3.61E-06	3.69	0.005
		Carboxylic acid metabolic process	19	10.56	3.61E-06	3.69	0.005
		Organic acid metabolic process	19	10.56	3.71E-06	3.68	0.005
		Cellular lipid metabolic process	19	10.56	3.93E-06	3.66	0.006
		Response to hydrogen peroxide	6	3.33	4.71E-06	23.23	0.007
	CC	Cytoplasm	114	63.33	4.81E-13	1.68	1.11E-10
		Cytoplasmic part	85	47.22	1.37E-11	1.94	3.14E-09
		Mitochondrion	37	20.56	6.64E-08	2.67	1.53E-05
		Intracellular part	128	71.11	5.25E-06	1.27	0.001
		Intracellular	130	72.22	2.52E-05	1.23	0.006
	MF	Oxidoreductase activity	29	16.11	2.67E-10	4.12	1.02E-07
		Oxidoreductase activity, acting on CH-OH group of donors	13	7.22	2.96E-09	10.68	1.13E-06
		Oxidoreductase activity, acting on the CH-OH group of donors, NAD or NADP as acceptor	6.67	6.67	1.19E-08	10.91	4.56E-06

*BP*: Biological process; *CC*: Cellular component; *MF*: Molecular function.

### IHC suggested irradiation-induced Leydig cell hyperplasia

**Figure 3:**
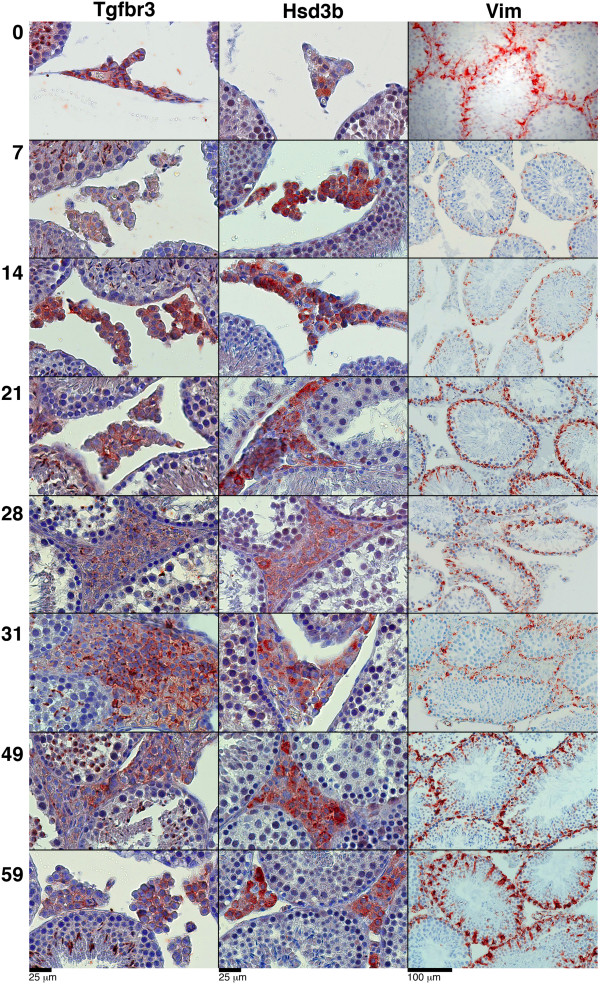
**IHC staining of the testis tissue pi with Leydig and Sertoli cell markers.** Tgfbr3 and Hsd3b were used as Leydig cell markers and Vimentin (Vim) was used as a Sertoli cell marker. Indications of Leydig cell hyperplasia are observed at pi day 21–31, whereas the Sertoli cells do not seem affected. A detailed description of the pictures is found in the text.

Large expression changes were observed in the somatic cell cluster. We expected this to be caused mainly by changes in cellularity, but we decided to use IHC to investigate whether histological changes contributed to the observed changes in expression. We stained sections of the irradiated testis tissue with the following somatic cell-specific markers: two Leydig cell markers: Tgfbr3 and Hsd3b; a PTM cell marker: SMA; and a Sertoli cell marker: Vimentin (Figure 3).

In control material (day 0; Figure 3), Tgfbr3 was expressed in the cell membrane of the majority of Leydig cells. Already at pi day 7 the Leydig cell membrane reaction appeared disturbed, incomplete or became cytoplasmic. In addition, the shape of the Leydig cells became more round and the close configuration apparent in the control material seemed to be lost. At pi day 21 some membrane reaction reappeared, but was lost again day 28–31 where hyperplasia of the Leydig cells was pronounced. During pi day 49–59 some of the Leydig cell membrane reactions was restored, but Tgfbr3 was only expressed at the membrane in about half of the Leydig cells at pi day 59 while the rest either showed a cytoplasmic reaction or no reaction at all.

Hsd3b was expressed in the Leydig cell cytoplasm in the control (day 0; Figure 3). Only a few Leydig cells reacted strongly, whereas the majority reacted moderately to faint or were negative. Again, already from pi day 7, this picture changed with the vast majority of the Leydig cells now reacting strongly to the antibody and although the intensity of the reaction gradually declined, it was still elevated at pi day 59. Staining with Hsd3b indicated an increase in Leydig cell numbers at pi day 21–28, but the number gradually returned to almost pre-irradiation levels at pi day 59.

Vimentin showed a specific staining in Sertoli cells in the control material (day 0; Figure 3). The staining remained in Sertoli cells throughout recovery, but the intensity and amount of staining seemed to decrease as specific germ cells disappeared. Already from pi day 7 where only spermatogonia are absent from the testis, the amount of staining was reduced, and it remained reduced until pi day 49 where the intensity and amount of staining was back to control levels. In addition, the classical extension of Sertoli cell cytoplasm towards the centre of the tubuli was mainly observed at day 0 and after pi day 49. Although the staining was reduced as distinct germ cell populations were missing, we did not find any indications of cell death among the Sertoli cells (deduced from the Vimentin staining). Neither did we find any histological changes pi in the PTM cell population by staining with SMA (results not shown).

**Figure 4:**
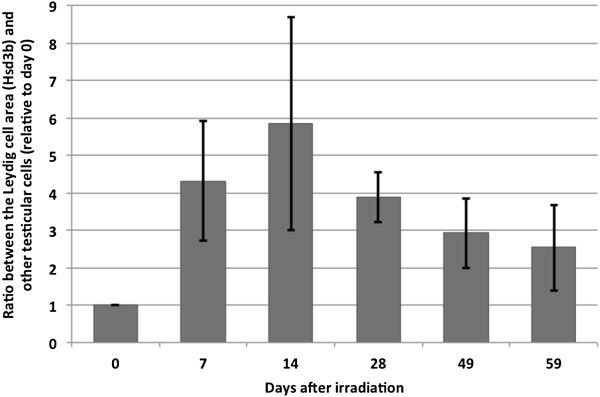
***In silico *determination of Leydig cells relative to other testicular cells pi.** Areas covered by Leydig cells highly increased following irradiation compared to areas covered by other testicular cells. The peak is observed at pi day 14 where the ratio decreased again.

Nevertheless, IHC staining of the testis tissue pi with the Leydig cell markers Tgfbr3 and Hsd3b suggested that some Leydig cell hyperplasia occurred after low dose irradiation. To confirm the hyperplasia pi, we quantified the ratio of the areas occupied by Leydig cells relative to the areas covered by other testicular cells. We found that the area of Leydig cells increased after irradiation and gradually decreased towards pre-irradiation levels as the testis was repopulated by germ cells (Figure 4). This further underlined that the number of Leydig cells seemed to be affected by low dose irradiation that may have caused Leydig cell hyperplasia.

## Discussion

### Cell-specific transcript clusters

We identified five unique transcript clusters with an unsupervised cluster analysis of the transcripts that changed in expression during recovery from irradiation. The expression changes observed in this study were caused by a combination of changes in cellularity and cells changing transcription pattern due to their differentiation towards spermatids and spermatozoa - both events change the total RNA pool. It further complicates the separation of the clusters that cells have a mixed cellular expression of many of the genes that are specific for spermatogenesis e.g. a relatively low expression that initiates in spermatogonia that is followed by a large increase in expression in spermatocytes (for example *Deleted in azoospermia-like* (*Dazl*) [22]).

We identified two spermatid clusters, one associated with early spermatids and a novel cluster associated with late spermatids. We anticipate that the transcripts in the early spermatid cluster initially were expressed in late pachytene or diplotene spermatocytes just before they entered meiosis, but with their main expression in early spermatids. It has previously been hypothesized that DNA is inaccessible to transcription during meiosis but also that a large group of mRNAs needed in early spermatids are expressed in the late spermatocyte stages [16,23]. Transcription is reinitiated in early spermatids [16] resulting in a massive wave of transcriptional activity immediately after meiosis before the chromatin is condensed and transcription is silenced during spermiogenesis [24,25]. Thus, the late spermatid cluster that we identified likely includes transcripts that are initially expressed during this wave of transcription in early spermatids.

The somatic cell cluster included markers specific for Leydig, PTM and Sertoli cells, suggesting that this cluster contained transcripts expressed by all the somatic cells in the testis. The somatic cells further comprise the microvasculature, lymph vessels, nerve fibres and connective tissue [26] and transcripts expressed by these cells are properly also present in the somatic cell cluster. From inspection of the expression profile of the somatic cell cluster it seemed that the somatic cells underwent large expression changes pi. However, the majority of the somatic cells did not seem to be affected by the irradiation and the gene expression changes detected in the somatic cell cluster were most likely caused by changes in cellularity. Since pachytene spermatocytes physically are the largest and most RNA-rich germ cells in the testis [10,26] their disappearing leads to the apparent expressional upregulation of genes from the somatic cell cluster where especially RNA from Sertoli cells comprise a larger part of the RNA from the testis.

We tried to separate more than five cell clusters from this data set. Yet, some of the original clusters just separated into subclusters with very similar expression patterns. The matured spermatozoa have a highly condensed transcriptionally inactive chromatin [27] and it is therefore reasonable that we did not identify any cluster(s) originating from the later germ cell stages.

### Gene set enrichment analysis

We performed GSEA of the subsets of the transcripts in each cluster to gain knowledge of their protein function. The analyses supported the association of the clusters with the cell types. However, the data set was complex and other cells than those specifically associated to the cluster may contribute to the pool of transcripts in each cluster.

The transcripts in the spermatogonia cluster were enriched for transcripts involved in mRNA processing, spliceosome and nucleus, in agreement with the transcriptional regulation involved during the numerous divisions and differentiations undergone by spermatogonia [28]. The transcripts in the spermatocyte cluster were enriched in male gamete generation, spermatogenesis and cell cycle process, which fit well with the meiotic processes in spermatocytes. Both spermatid clusters were enriched in spermatogenesis. The early spermatid cluster was additionally enriched in acrosomal vesicle, which agrees with the acrosome being formed during the maturation of early to late spermatids and further to spermatozoa [29]. The transcripts in the late spermatid cluster were in addition enriched in serine-type peptidase activity and especially one serine protease, acrosin, has been identified as an important enzyme in the acrosome [30].

The very diverse functions of the somatic cells were also observed in the GSEA where the enriched categories among others included lipid metabolic process, response to oxidative stress and lipid biosynthetic process. Since cholesterol is the basis for steroidogenesis in Leydig cells, lipid metabolism is essential for steroid production [31-33]. Both the many cell divisions in the germinal tissue and Leydig cell steroidogenesis require large amounts of oxygen. The testis is poorly vascularized and therefore vulnerable to oxidative stress and consequently have several defence mechanisms where Sertoli cells superoxide dismutase is important [34]. Thus, the results from the GSEA on the transcripts in the somatic cell cluster represented both Leydig and Sertoli cell functions.

### Low dose irradiation may cause Leydig cell hyperplasia

IHC validation of the two Leydig cell markers, Tgfbr3 and Hsd3b, showed that low dose irradiation affected the appearance of the Leydig cells with indications of Leydig cell hyperplasia around pi day 21–31. Previously published studies reported that human Leydig cells are preserved even after high dose irradiation up to 20 Gy, but that the endocrine function of the cells are affected also at lower doses [35-38]. In this study, the testes were irradiated with 1 Gy in a single dose, which is a low dose even in mice compared to the clinical doses of 16–20 Gy used to treat human testicular cancer [39].

Leydig cell hyperplasia has also been observed in men with fertility problems and testicular cancer [40]. The proliferation might arise due to a decreased testosterone production by the Leydig cells pi, which will induce increased LH production that may stimulate proliferation of pre-Leydig cells and their differentiation to Leydig cells. This is also the clinical observation upon irradiation of human testis harbouring carcinoma *in situ*[41] and in rats with Sertoli cell-only testes [42]. However, it is not clear if a dose of 1 Gy leads to reduced testosterone production and increased LH level, although higher irradiation doses lead to changes in testosterone and LH levels [43]. Also, the presence of Follicle-stimulating hormone (FSH) receptors in Leydig cells could suggest that the proliferation could be a response to decreased inhibin production caused by the absence of germ cells, which leads to increased FSH levels that may induce proliferation of pre-Leydig cells [44]. Further, the GSEA showed that the transcripts in the somatic cell cluster were highly enriched in lipid and steroid biosynthetic processes (see Additional file 11: Table S10). This enrichment can either be caused by an increased activity of the remaining Leydig cells or by an increased number of Leydig cells. From our results it seems to be caused by an increased number of Leydig cells. Alternatively, the reduced number of germ cells in the tubuli will likely result in a reduced size of the tubuli, and this might alter the ratio between tubuli and interstitial areas.

IHC staining of Vimentin specific to Sertoli cells did not show any histologically changes during recovery from low dose irradiation. Earlier studies found Sertoli cell death following irradiation, but these were all studies that used higher irradiation doses [45,46]. SMA staining of PTM cells did not suggest any histological changes in the PTM cells.

## Conclusion

In this study, we investigated the molecular and cellular changes following re-establishment of spermatogenesis pi by analysis of the testicular transcriptome. We identified five clusters each representing a profile of a specific type of cell(s) during recovery from irradiation. We associated the five clusters with: spermatogonia, spermatocytes, early spermatids, late spermatids and somatic cells, respectively. We observed large expression changes in the somatic cell cluster during the time series. Therefore, we investigated if changes in histology, in addition to changes in cellularity, contributed to this observation. IHC staining of Leydig cell-specific markers suggested Leydig cell hyperplasia at pi day 21–31, which was further supported by *in silico* quantification of the relative area occupied by Leydig cells.

## Competing interests

The authors declare that they have no competing interests.

## Authors’ contributions

HL designed the study; MT conducted irradiation experiments; MDD and KCB performed the microarray experiments; KCB and HBN performed the data analysis; JEN performed the immunohistochemistry; KA performed the *in silico* determination of Leydig cells; KCB, HBN, SB, KA and HL interpreted the results; all authors contributed to the writing of the manuscript. All authors read and approved the final manuscript.

## Supplementary Material

Additional file 1: Figure S1 An example of testis tissue sections stained with the Leydig cell-specific Hsd3b (red) and counter stained with Meyers haematoxylin (blue). Red stain was assigned a red colour, blue stain a blue colour and the background assigned white. *In silico* separation of the colour space using a Baysian algorithm separated blue from red stain and hence Leydig cells from all other cells. This figure shows the layer of assigned colours with different transparency on top of the original image. Sections from day 0 and pi day 7, 14, 28, 49 and 59 were used for *in silico* quantification of the Leydig cells versus the other testis cells. Click here for file

Additional file 2: Table S1 Gene names and identifiers of the 109 transcripts assigned cluster 1 based on the cluster analysis of the subset of 988 transcripts. Click here for file

Additional file 3: Table S2 Gene names and identifiers of the 164 transcripts assigned cluster 2 based on the cluster analysis of the subset of 988 transcripts. Click here for file

Additional file 4: Table S3 Gene names and identifiers of the 246 transcripts assigned to cluster 3 based on the cluster analysis of the subset of 988 transcripts. Click here for file

Additional file 5: Table S4 Gene names and identifiers of the 273 transcripts assigned cluster 4 based on the cluster analysis of the subset of 988 transcripts. Click here for file

Additional file 6: Table S5 Gene names and identifiers of the 196 transcripts assigned cluster 5 based on the cluster analysis of the subset of 988 transcripts. Click here for file

Additional file 7: Table S6 All Gene Ontology categories from the gene set enrichment analysis performed using DAVID on cluster 1 - spermatogonia. Click here for file

Additional file 8: Table S7 All Gene Ontology categories from the gene set enrichment analysis performed using DAVID on cluster 2 - spermatocytes. Click here for file

Additional file 9: Table S8 All Gene Ontology categories from the gene set enrichment analysis performed using DAVID on cluster 3 – early spermatids. Click here for file

Additional file 10: Table S9 All Gene Ontology categories from the gene set enrichment analysis performed using DAVID on cluster 4 – late spermatids. Click here for file

Additional file 11: Table S10 All Gene Ontology categories from the gene set enrichment analysis performed using DAVID on cluster 5 – somatic cells. Click here for file
