# Supplementary figures and images for: Transcriptome profiling of mice testes following low dose irradiation
Source: Reprod Biol Endocrinol. 2013 May 28;11:50. doi: 10.1186/1477-7827-11-50 (PMC3672050; doi:10.1186/1477-7827-11-50)

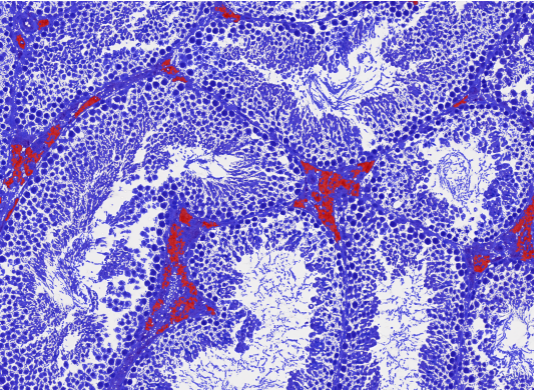

50% transparent

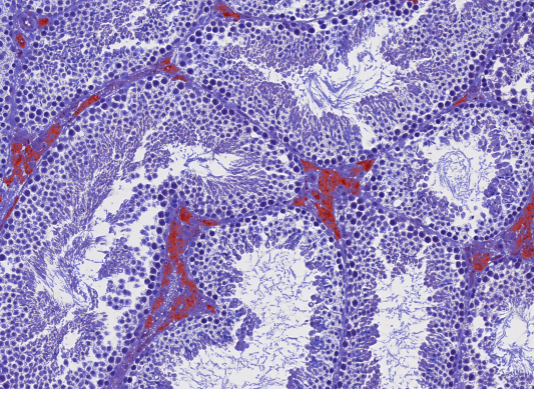

75% transparent

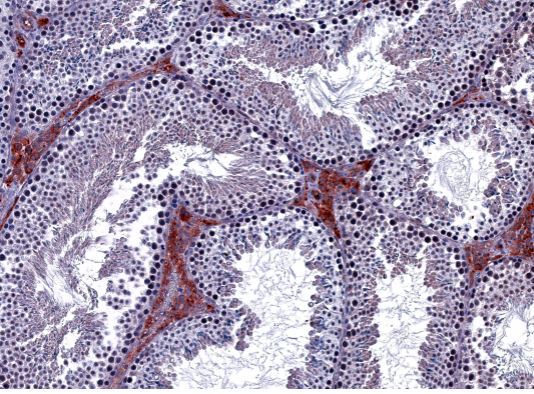

100% transparent

Supplement: Additional file 1: Figure S1 — An example of testis tissue sections stained with the Leydig cell-specific Hsd3b (red) and counter stained with Meyers haematoxylin (blue). Red stain was assigned a red colour, blue stain a blue colour and the background assigned white. In silico separation of the colour space using a Baysian algorithm separated blue from red stain and hence Leydig cells from all other cells. This figure shows the layer of assigned colours with different transparency on top of the original image. Sections from day 0 and pi day 7, 14, 28, 49 and 59 were used for in silico quantification of the Leydig cells versus the other testis cells. [file 1477-7827-11-50-S1.pdf]
